# Nanoparticles improved pheophorbide-a mediated photodynamic therapy for cancer

**DOI:** 10.1007/s10103-025-04320-2

**Published:** 2025-02-07

**Authors:** Nosipho Fakudze, Heidi Abrahamse, Blassan George

**Affiliations:** University of Johannesburg, Johannesburg, South Africa

**Keywords:** Pheophorbide-a, Photosensitizer, Nanoparticles, Photodynamic therapy, Cancer

## Abstract

The increased cancer incidence and mortality rates have made researchers continue to explore different types of effective and less toxic cancer therapies. Photodynamic therapy (PDT) is an alternative cancer treatment modality with reduced side effects. It is comprised of three components, a photosensitizer, molecular oxygen and light. Researchers have been exploring third generation photosensitizers that overcome existing photosensitizer limitations such as hydrophobicity, accurate targeting and photosensitivity. Pheophorbide-a is a chlorophyll product currently being explored in a number of in vitro, in vivo and in silico studies as an ideal photosensitizer for breast, prostate, lung, oral squamous cell carcinoma, gastric, osteosarcoma and cervical cancers. These in vitro, in vivo and in silico studies have shown the probable cell death pathways in different cell lines and how advancement in using nanocarriers has improved cancer cell killing effect after pheophorbide-a mediated PDT. The pharmacokinetics have elaborated on the biodistribution and tissue disposition of pheophorbide-a in this review. In summary, we offer our viewpoint on PDT in the context of cancer management, and we believe that this article will shed new light on the role of pheophorbide-a in PDT for cancer.

## Introduction

Cancer can be described as the aberrant growth of cells due to numerous changes in gene expression that disrupt the normal balance between cell proliferation and death [1, 2]. The recent global cancer statistics showed 9.7 million deaths and 20 million estimated new cases were reported in 2022. The cancer treatment modalities include radiation therapy, surgery, targeted therapy, photodynamic therapy (PDT) and chemotherapy. The type and stage of cancer are among the many variables that influence the recommended therapeutic modalities [3–5].

PDT is an alternative approved therapy that combines light, molecular oxygen, and photosensitizer (PS). PDT has been used for the treatment of a number of cancers, including lung, skin, bladder, oesophagus, T-cell lymphoma, prostate, bladder, and breast [6, 7]. The photosensitizing agent is introduced intravenously or topically; localizes in the tissues and is then activated by light. The PS does not react with biomolecules in and of itself; instead, exposure to light of specific wavelength produces reactive oxygen species (ROS) such as singlet oxygen, hydrogen peroxide, hydroxyl radical, and superoxide radical, when energy is transferred from light to molecular oxygen. These photoproducts activate metabolic reactions that cause cellular damage or death [8].

PS can be classified under first-, second- and third-generation. Each PS class has certain characteristics that are advantageous but have limitations that lead to their improvement and move from first to third generation [9]. In 1841 when Scherer isolated hematoporphyrin, this was the starting point of the discovery of functionality of PS and is the backbone of PS in present times. First-generation PS are commonly termed porphyrins. Porphyrin derivatives synthesised after the late 1980s are referred to as second generation PS. In third generation, PS take pharmaceuticals that are already on the market and alter them with biologic conjugates, built-in photobleaching capabilities, antibody conjugates, and more [10–12]. Second-generation PS pheophorbide-a is a bacteriochlorin based chlorophyll a derivative with cytotoxic activity. Its drawbacks include poor water solubility and lack of specificity in drug delivery [13]. These drawbacks have been addressed by the use of nanotechnology that has capabilities of targeting tumour cells [13, 14]. The aim of this review is to explore the photosensitizer pheophorbide-a, its pharmacokinetics, pharmacodynamics, and studies with and without nanotechnology integration.

## Pheophorbide-a

**Fig. 1:**
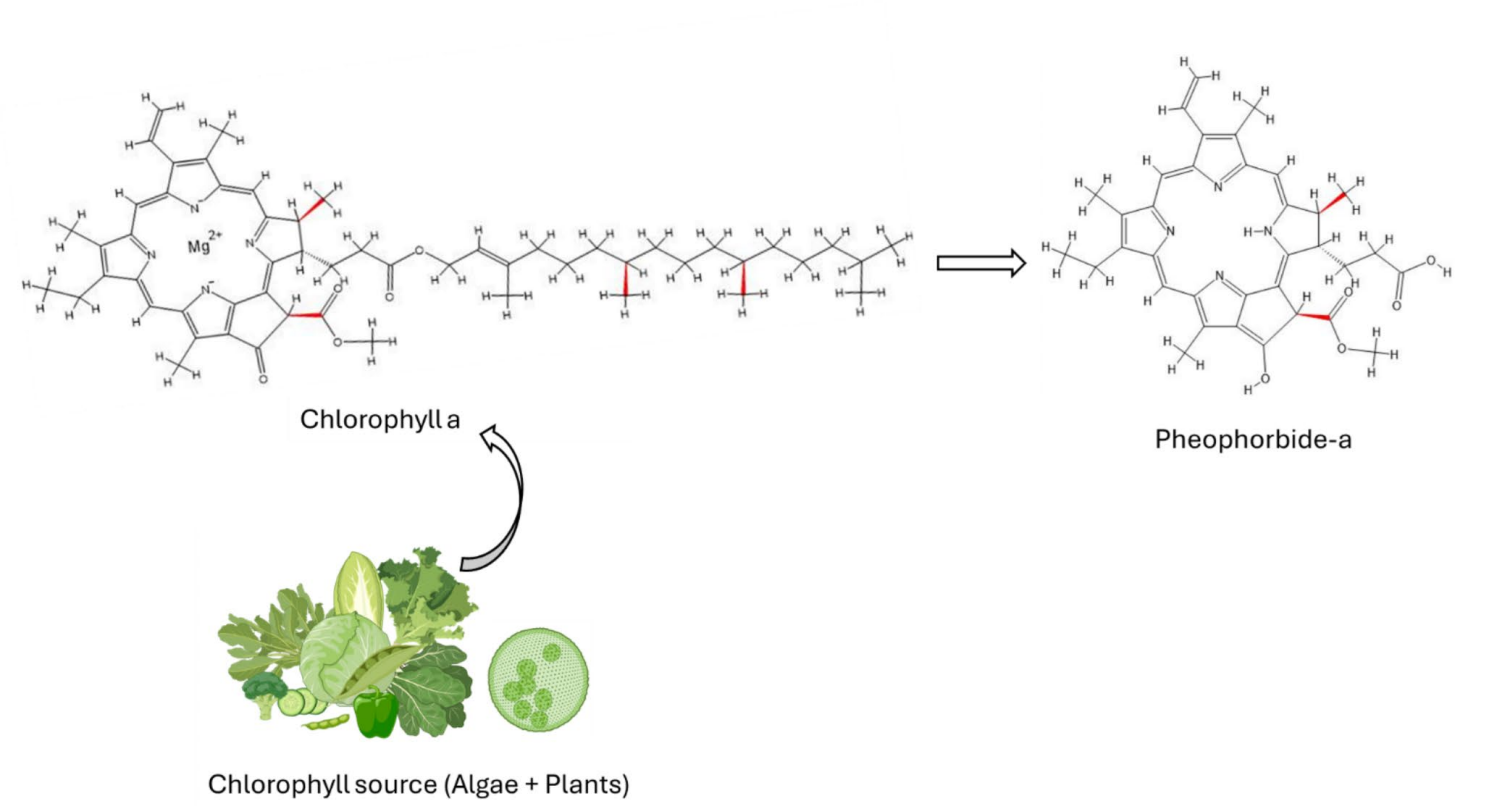
Synthesis of pheophorbide-a

Pheophorbide-a has been researched significantly due to its photosensitising capabilities since 1996 [15–18]. Studies have shown its antioxidant, anti-inflammatory, and phototoxic capabilities [15, 16, 19]. It is an anionic dye found naturally and a catabolic product of chlorophyll [15]. Algae and higher plants are responsible for the production of pheophorbide-a through the dephytylation and demetallation of chlorophyll a (Fig. 1). This process uses magnesium-dechelatase and chlorophyllase to mediate the catabolic transformation [17]. The tetrapyrrolic microcycle that makes up pheophorbide-a has four methyl substituents, an ethyl, a methoxycarbonyl, a propionyl, and a vinyl [17]. Chlorophyll a UV-vis spectra show peaks at 500 to 700 nm (Q-band), with a relatively high Q-band at 670 nm and Soret band at 390 nm [17]. One of the major benefit of using pheophorbide-a as a photosensitizer in PDT is that, light irradiation in anoxic environment causes the cleavage of nucleic acids by pheophorbide-a radical species created when labile hydrogen atom of the five-member isocyclic ring are photo-removed [15].

## Nanoparticles

A material having internal or surface structure, or external dimensions at the nanoscale, is referred as nanomaterials [20]. Nanomaterials are used in different applications such as water treatment, medicine, energy storage, agriculture and as a catalyst due to their unique characteristics [21]. Nanoscale can be defined as sizes ranging from 1 to 1000 nm [20] or 1–100 nm [22]. Nanoparticles used as drug delivery systems improve drug solubility, control drug release properties, direct drug molecules to specific locations, and administer multiple medications at once [9]. In this review we shall be exploring passive and active PS nanocarrier treatment modalities for improved drug delivery and overall treatment effects compared to none nanocarrier system options.

### Passive PS nanocarrier systems for PDT of cancer

**Fig. 2:**
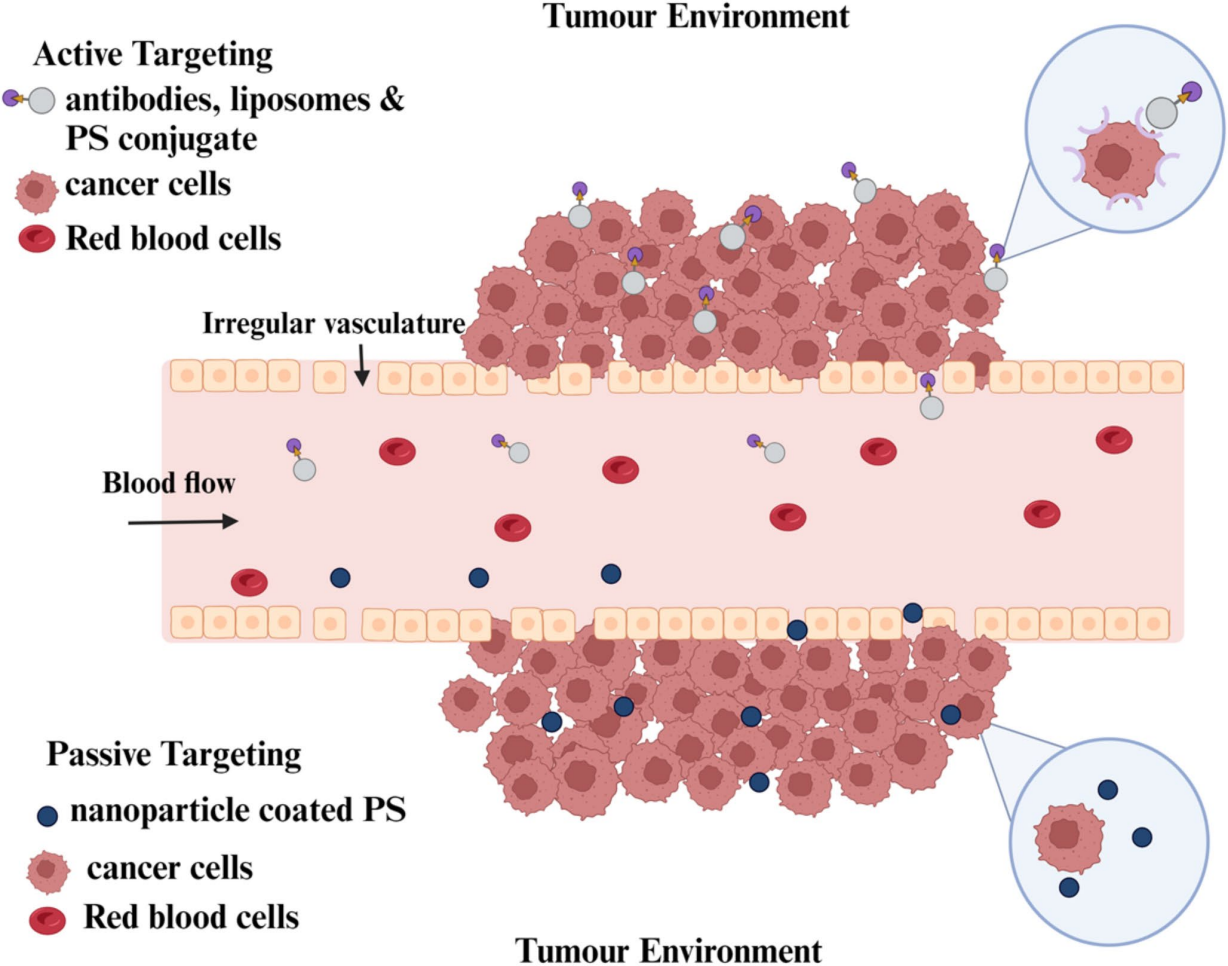
Passive and active targeting to enhance photodynamic therapy outcome. NPs can be actively targeted to improve NP accumulation and cellular uptake through receptor-facilitated endocytosis, and passively targeted to capitalize on EPR effect

This nanocarrier system utilizes the deteriorated tumour environment for PS administration. The environment consists of poor lymphatic drainage, wide intercellular spaces, and activated endothelial cells. Nanocarriers used for tumours are retained in this type of environment due to the absence or dysfunction of lymphatic vessels, resulting in increased vascular retention [23]. Limitation of passive targeting include absence of content release on demand (Fig. 2) [24]. One disadvantage of using low molecular weight drugs (40 kDa) in passive targeting is early drug diffusion which leads to drug entering the bloodstream away from target site [25]. This size-dependent enhanced permeability and retention effect varies depending on the patient and the type of tumour. The high fluid pressure at its interstitial space, endosomal escape, intricacy in the extracellular matrix, relative hypoxia, and difficulty in tumour penetration due to varying endothelial gaps all have an impact on enhanced permeability and retention effect [25].

### Active PS nanocarrier system for PDT

The nanocarriers used in the active targeting strategy have tiny ligand molecules on their surface that bind to a particular receptor and hold it there while also allowing the diseased cells to actively absorb it. This method is the most commonly used and has a high degree of selectivity when it comes to binding the target site. When identifying the overexpressed protein receptors in diseased cells that are absent in healthy cells, the binding ligand should be specific [25]. These biological ligands frequently attach to particular receptors on the surface of the target cells, increasing the drug-containing nanoparticles’ cellular uptake as well as the effectiveness of treatment (Fig. 2) [26]. Small molecules, antibodies, lectins, lipoproteins, carbohydrates, peptides, nucleic acids, hormones, glycoproteins (transferrin), growth factors, polysaccharides, and folic acid are some examples of the targeting ligands [25, 27]. Due to their high surface-to-volume ratio, the nanocarriers can also be tailored to target different moieties. Since active targeting can lessen multi-drug resistance and prevent off-site drug delivery, it is preferable compared to passive targeting [27].

## Pheophorbide-a-Mediated PDT studies

Pheophorbide-a has been used as an ideal PS for numerous studies in the past decade. Both in vitro and in vivo studies have shown significant cancer killing affect through mostly activation of apoptotic pathways. Research on its effects and mechanism of cell death has been elaborated in the following sections and summarized in Table 1.

**Table 1:** Summary of in vitro and in vivo studies on pheophorbide-a

In vitro studies				
Cell line	**Cancer type**	**Cell death mechanism**	**Dosage**	**Reference**
LNCaP and PC3	Prostate cancer	ROS production and subsequent oxidation induction of membrane lipids.	0.25 µM	[28]
LNCaP	Prostate cancer	ROS production causing ER stress and cell cycle arrest at G_0/_G_1_	5 µM	[29]
MCF-7-doxorubicin resistant	Breast cancer	Photokilling effect	2.5 µM	[18]
MDA-MB-231	Triple Negative Breast cancer	ROS production of *NRF2*-knockdown cells	0.025 µg/mL	[30]
HuO9	Osteosarcoma cells	Intrinsic pathway activation via mitochondria	28 µmol/L	[31]
HeLa	Cervical cancer	Chemotoxicity and phototoxicity	2 µM	[32]
In vivo studies				
AT-84	Murine oral squamous cell carcinoma	Decrease in PCNA expression in histopathological exams, and apoptosis via activation of intrinsic pathways	10 mg/kg	[33]
N/A	Mouse papilloma’s	Partial lesion removal and apoptosis via cleavage of caspase 3	Topical application: 100 µg in 100 µL of acetone per mouse	[34]
BALB/c	Mouse breast cancer	Activation of immune response	5 mg/kg	[35]

### In vitro studies

Androgen sensitive (LNCaP) and insensitive (PC3) prostate cancer cell lines showed reduced cell viability of approximately 90% at 0.25 µM of pheophorbide-a with signs of apoptosis through increased levels of caspase 3 and 8, PARP expression and Bax proteins and reduced expression of caspase 9 and Bcl-2 [28]. In another study pheophorbide-a mediated PDT on LNCaP prostate cells showed a significant cell death at 5 µM with cell cycle arrest at G_0/_G_1_ phases after irradiation 30 min and increased levels of reactive oxygen species [29]. A study carried out on doxorubicin drug resistant breast cancer cells (MCF-7) showed reduced cell viability at 2.5 µM of pheophorbide-a at approximately 85% with cell damages shown through morphological assays [18]. NRF2-knockdown breast carcinoma MDA-MB-231 cells were used to show NRF2 signalling as a possible molecular factor of (0.025 µg/mL) pheophorbide-a mediated PDT. Stable NRF2 knockdown cells exhibited increased ROS and singlet oxygen levels after irradiation (0.6 J/cm^2^), as well as enhanced cytotoxicity and apoptotic/necrotic cell death [30]. HuO9 osteosarcoma cells were treated with Na-pheophorbide-a and then irradiated at 0.1–10 J/cm^2^, 150 mW, 664 nm. Study showed a cytotoxic effect on the osteosarcoma cell line with an increase in the number of cells positive for TUNEL staining and an increase in caspase-3, -8, and − 9 activity. An apoptotic cell death mechanism was seen due to primary activation in mitochondrial caspase − 9 and − 3 pathways [31]. The combined treatment of HeLa cells with doxorubicin and pheophorbide-a mediated PDT results showed enhanced cytotoxicity at lower dosage of the chemotherapeutic medication. Treatment dosage was at 0.2 µM of doxorubicin and 2 µM of pheophorbide-a (6.4 J/cm^2^). Cytotoxicity enhancement may be due to the different subcellular localizations of PS and drug, their targets, and modes of action [32].

### In vivo studies

The treatment of murine oral squamous cell carcinoma using pheophorbide-a mediated PDT showed a decreased in PCNA expression in histopathological exams, and activation of apoptosis with decrease of bcl-2 and caspase 3 cleavage was observed in immunoblot [33]. Induced mouse papilloma’s were treated with pheophorbide-a when irradiated showed results of 17.5% of lesion removal in the first week and 31.7% removal after a second exposure of treatment [34]. The treatment using PDT and cyclic RGD modified liposomal loaded with anti-PD-L1 (programmed cell death ligand 1) and pheophorbide-a on mouse breast cancer showed an enhanced antitumour immune response [35].

### Pheophorbide-a mediated PDT with targeting moieties

Over the past few decades, there has been an increase in the use of targeted delivery strategies for anticancer drugs with successful results in preclinical trials [36]. Actively targeted drugs may exhibit improved treatment efficacy because they accumulate in the area of interest (tumour site) and occur less in healthy tissue [37]. Targeting takes advantage of the high affinity of cell-surface to targeted ligands, in order to specifically retain and uptake a drug by the targeted disease cells [38].

#### In vitro studies

Pheophorbide-a has drawbacks such as its hydrophobic nature and this has been reviewed by researchers for better effectiveness in cancer therapy through the use of nanoscale systems which greatly improve PS solubility in biological media, stability, and selectivity of PS build-up in cancer cells [39]. In a study that compared free pheophorbide-a and poly lactic-co-glycolic acid nanoparticles containing pheophorbide-a, with the surface containing polyethylene glycol and folate showed improved retention period in the body, which led to an EPR effect that increased pheophorbide-a accumulation in tumour tissue by more than ten times. This compound showed rapid uptake on MKN28 human gastric cancer cells and remained stable for more than a week. When cells were irradiated (670 nm, 0.3 W, 0.75 J) they showed significant cytotoxicity [40]. In another study they showed that the incorporation of paclitaxel prodrug and pheophorbide-a loaded nanoparticles inhibited the photosensitizer’s aggregation, resulting in a greater level of ROS and singlet oxygen generation. On SK-OV-3 cells a 30 times dose reduction of paclitaxel and a 3 times dose reduction of pheophorbide-a was seen compared to nanoparticle complex, thereby potentially reducing the overall systemic toxicity while maintaining the tumour efficacy. Cell death mechanism shown in this study is predominantly through apoptosis using paclitaxel while pheophorbide-a medicated PDT showed less effect on SK-OV-3 and MDA-MB-231 cells at 0.26 µM and 0.35 µM doses (pheophorbide-a allowed for reduced PS aggregation and reduced systemic toxicity through reduction in dosage of treatment) [41].

#### In vivo studies

Casein micelle loaded with hydrophobic (5 mg/kg) pheophorbide-a was used to treat SCC7 tumour cells in mice. Conjugate demonstrated excellent stability over four months in aqueous conditions without aggregating. Intravenous injected conjugate was rapidly absorbed into SCC7 tumour cells at 2-fold compared to free pheophorbide-a and when irradiated (671 nm, 50 W) caused cancer cell death. The tumour growth was suppressed with no obvious adverse effects [42]. Mice injected with colorectal cancer HT-29 cells upon treatment with (7.5 mg/kg) pheophorbide-a loaded nanofibrils showed improvements in dosing, ROS generation, and photothermal conversion efficiency. Pheophorbide-a’s water solubility and targeting capability improved once it was aggregated into the nanostructure [43].

### In silico studies on Pheophorbide-a

The use of in silico methods are for better understanding and forecasting how drugs impact biological systems which can sequentially enhance clinical usage, prevent undesirable side effects, and direct more effective treatments [44]. In silico techniques (AutoDock Vina, Schrödinger molecular modelling) have been used to identify target proteins for certain cancer cells to plan treatment modalities. In a study that evaluated the effectiveness of *Chrysanthemum cinerariifolium* stem ethanol extract against oral squamous cell carcinoma. Metabolic profiling showed extract contain 8.05% pheophorbide-a. The in silico analysis revealed high hydrogen boding number between pheophorbide-a at -9,4 kcal/mol to PI3K protein, showing the strongest binding affinity [45]. Another study evaluated the *Typhonium flagelliforme* extract against human lung cancer cell lines, and revealed the presence of pheophorbides, phytol and its derivatives, hexadecanoic acid, and 1-hexadecene. The probable cause of cancer cell growth inhibition was due to 1-hexadecane and hexadecanoic acid binding to the switch II region of K-ras, and pheophorbide-a binding on K-ras thereby interfering with the interaction between the nucleotide exchange protein between SOS (son of sevenless) and K-ras [46]. In a study done by Souid and colleagues the anti-proliferative ability of *Ziziphus lotus* extracted and purified compounds like protopheophorbide-a was assessed against metastasized triple negative breast cancer cells (MDA-MB-231). Protopheophorbide-a caused apoptosis in these cells and showed the strongest inhibitory effect on cancer cell viability. Protopheophorbide-a’s docking screens revealed an in-pocket orientation of its pheophorbide-a scaffold within the ATP-binding cleft of both wild-type and mutant c-Met kinases. Protopheophorbide-a also displayed the highest virtual affinity toward the c-Met tyrosine kinase [47].

### Pharmacokinetics and pharmacodynamics of Pheophorbide-a

Pheophorbide-a pharmacokinetics show poor gastrointestinal absorption, low penetrability to glycoproteins, and no ability to cross the blood–brain barrier. Its solubility was determined to be soluble, at a value between 0 and 2 lipophilicity. Pheophorbide-a shows 4 H-bond acceptor, 3 H-bond donor, cLog p-value of 3.82, topological surface area of 133Å, molecular weight of 592.68 and a computed human lethal dose at 40 mg/kg of body weight [16, 48]. Uchendu et al. investigated pheophorbide-a bioavailability and found it at 56% (using SwissADME). They further theorized that it would be found in the bloodstream at a significant level thereby causing therapeutic effect. Moreover, no toxicity was documented for pheophorbide-a at doses up to 2 g/kg in mice; a maximum dose of 1000 mg/g was recommended [49].

Biodistribution and tissue disposition of pheophorbide-a was found in the blood, kidney, gallbladder and especially the liver [50–52]. Mechanistic investigations demonstrated that although HPPH a derivative of pyropheophorbide-a was a potent mediator of vascular photodamage, it induced minimal direct toxicity to tumour cells. After administering 1 mg of HPPH intravenously per kilogram, pharmacokinetic analyses demonstrated a biexponential decline over time, with plasma half-lives of β and α being 21 and 0.69 h, respectively. The main method of elimination was faecal excretion. The liver exhibited the greatest long-term retention and the highest levels of HPPH. The liver, adrenal glands, lung, spleen, kidney, bladder, heart, eye, skin, pancreas, muscle, testes, and brain were the organs in decreasing order of absorption [50, 53]. In a study conducted using mice, it was discovered that some absorption of pheophorbide-a was facilitated through scavenger receptor B type I (SR-BI) protein with accumulation in the liver [54].

## Conclusion

Cancer remains one on the leading causes of death worldwide and its incidence rate continues to increase and is projected to almost double by 2050. It has been projected that a 77% increase in new cancer cases will be seen, which is approximately 35 million cases compared to recorded 20 million in 2022 [55]. In this review, tetrapyrrolic based compound, pheophorbide-a has been explored for their effectiveness in the different malignancies with and without targeting moieties. The mechanism of cell death seen through the explored studies showed, apoptotic cell death, antitumour immune response, intrinsic and extrinsic cell death activation through the various malignancies. Pheophorbide-a mediated PDT with nanocarrier systems showed improved efficiency in treating the different cancers, from improved water solubility, targeting, and stability. In exploring the different cell death mechanism and improved efficiency of pheophorbide-a mediated PDT in the in vitro,* in vivo and in silico* studies, the gap to preclinical trials can be bridged and potentially reduce the disadvantages seen when moving to human trials. In using methods as mentioned above, clinical translational research downfalls such as poor hypotheses, unclear preclinical models, irreproducible data, statistical errors, and the clinical relevance of basic research can be reduced/improved. These techniques can better assist with understating drug efficacy, safety, screening and disease modelling techniques. This can then potentially pave the way for more preclinical research on pheophorbide-a and its derivatives as a potent PS.

## Data Availability

No datasets were generated or analysed during the current study.
